# Genotyping diagnosis of alpha-1 antitrypsin deficiency in Saudi adults with liver cirrhosis

**DOI:** 10.1097/MD.0000000000006071

**Published:** 2017-02-10

**Authors:** Noura Al-Jameil, Amina A. Hassan, Ahlam Buhairan, Rana Hassanato, Sree R. Isac, Maram Al-Otaiby, Basmah Al-Maarik, Iman Al-Ajeyan

**Affiliations:** a Collage of Applied Medical Sciences, Clinical Laboratories Department, King Saud University; b King Khalid University, Hospital, Riyadh, KSA.

**Keywords:** AAT, genotyping, liver cirrhosis, phenotyping

## Abstract

The acute phase protein alpha-1 antitrypsin (AAT) is mainly produced in liver cells. AAT deficiency affects the lungs and liver. We conducted a case-control study to define a valuable method for the proper diagnosis of alpha-1 antitrypsin deficiency (AATD), as well as the association of liver cirrhosis with AATD in Saudi adults.

Blood samples from 300 liver cirrhosis patients and 400 controls were analyzed according to serum AAT concentration, phenotyping, and genotyping. Nephelometry was used for AAT quantification, isoelectric focusing electrophoresis was used for phenotyping detection, and real-time PCR was used for genotyping to determine the Z and S deficiency alleles.

This study highlights the accuracy of using genotyping in addition to AAT quantification, since this technique has proven to be successful in the diagnosis of AATD for 100% of our cases. A significant deviation in AAT genotypes frequencies from the Hardy–Weinberg equilibrium in the adult cirrhosis group occurred due to a higher observed frequency than expected for the Pi ZZ homozygous genotype.

Pi ZZ in adults may be considered as the risk factor for liver cirrhosis. However, we could not establish this relationship for heterozygous AATD genotypes (such as Pi MZ and Pi SZ).

## 1 Introduction

Alpha-1 antitrypsin (AAT) is a protease inhibitor that is produced mainly by liver cells^[1]^ to inhibit neutrophil elastase for lung protection.^[2]^ AAT deficiency (AATD) is a hereditary disease, and a deficiency of plasma AAT leads to COPD and accumulation of the mutated protein molecules in the hepatocytes, which causes liver disease.^[3]^ AAT is encoded by the SERPINA1 gene, and it is highly pleomorphic with approximately 100 identified alleles. The native M allele variant exhibits full antiproteolytic activity. The Z and S alleles cause the most deleterious mutations in patients with AAT D, whereas the Z mutation is the most severe. The improperly processed Z gene product during synthesis of AAT, lead to aggregates in the liver, which results in liver disease.^[4]^ Overall, 30% of adult men with Pizz will acquire cirrhosis, whereas 10% of neonates will exhibit hepatitis.^[5]^ Additionally, people who have Pi SZ suffer from some liver diseases.^[6]^ The risk of AAT deficiency for liver disease in pediatrics medicines has been well- documented, but the association of AAT deficiency with liver disease in adults is less clear.

AAT deficiency is under-recognized despite Establishment of World Health Organization guidelines regarding testing criteria.^[7,8]^ Recent data from many countries have demonstrated that only 5% to 15% of homozygous individuals with this deficiency have been identified. Due to the lack of knowledge about the disease and suitable techniques, as well as low awareness about the disorder among physicians, the diagnostic delay typically exceeds 5 years, which has lead to an average age at diagnosis of approximately 45 years.^[9]^ This high average age confirms the necessity for efficient testing to increase detection of individuals who are at risk.^[10]^ Quantitative determination of serum AAT levels is not enough for the diagnosis of AAT deficiency because AAT is an acute phase reactant that may be increased due to of inflammation, which leads to misdiagnosis. Therefore, it is necessary to use other techniques such as phenotyping and genotyping.^[11]^

Although phenotyping identifies many different alleles (common and rare), this technique is time consuming and needs a high degree of technical skill to interpret the results. Additionally phenotyping cannot identify Pi Null alleles due to the absence of circulating protein and the need for special laboratory procedures. Often, errors in Pi phenotyping are due to poor sample quality or samples taken from the patient receiving AAT augmentation therapy.^[12]^ Genotyping of the SERPINA 1 gene, which reveals only the most common deficiency alleles Z and S, can miss 1 of the more than 30 rare alleles that cause a reduction in the AAT levels, but this genotyping test is important to include in the algorithms used to diagnose AAT deficiency because it decreases the need for costly IEF or sequencing assays.^[13]^

The purpose of this study was to determine the frequencies of deleterious AAT variants and their effects on adults Saudi patients with liver cirrhosis and to propose the appropriate technique for the early detection of AAT deficiency in at-risk individuals.

## 2 Material and methods

A case-control study was conducted with 300 adult patients who had liver cirrhosis and who received care at gastroenterology clinics collaborating with University Hospitals in different provinces of the Kingdom of Saudi Arabia, between June 2013 and June 2015. We also included 400 controls who matched the cirrhosis patients in age, sex, and socioeconomic levels and who were chosen from a pool of healthy blood donors at the same hospitals to minimize selection bias. Informed consent and approval from the Institutional Ethical Committee of each hospital at the different provinces were collected. Table 1 shows the sample cohorts and selection criteria. Blood samples for all tests were drawn before the biopsy procedure.

**Table 1:**
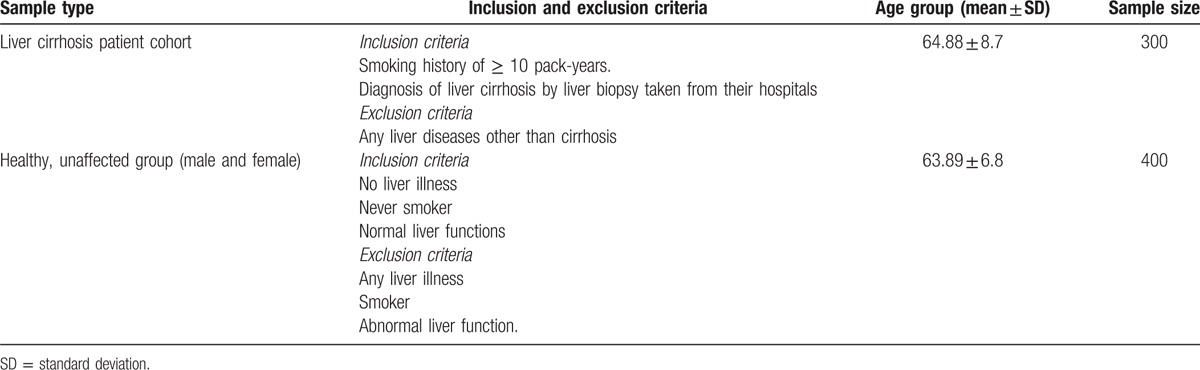
Sample cohorts and selection criteria.

Several parameters were recorded for each patients such as: serum AAT concentration, age, gender, smoking habits, biochemical tests (total billirubin, albumin, prothrombin time, aspartate aminotransferase, alanine aminotransferase, alkaline phosphatase and gamma glutamyl transferase), etiology of cirrhosis (which was viral if hepatitis B or C tests were positive, immunological if there were positive results for immunological markers or alcoholic if there was history of alcohol consumption), degree of liver function impairement by Child–Pugh classes, and presence and degree of oesophageal varices.

To calculate the Child–Pugh score 5 variables were used (i.e., ascites, encephalopathy, bilirubin, albumin, and prothrombin time). According to the increasing abnormality of these variables, values of 1, 2, or 3 were assigned to each, and the score was considered to be the sum of the 5 variables for patient. A Child–Pugh score less than 7 was considered class A, from 7 to 9 class B, and any score greater than 9 was class C.

Assessment of varices was carried out by an endoscopist, using a 4-grade classification. Varices not arising from the surrounding mucosa were recognized as grade I, varices smaller than 5 mm and less than one-third of the esophageal lumen were determined to be grade II, varices larger than 5 mm and more than one-third of the esophageal lumen were grade III, and grade IV varices were larger than two-thirds of the esophageal lumen. Samples with a mosaic pattern and speckled red spots of the mucosa were described as portal hypertensive gastropathy.

Nephelometric determination of serum AAT levels was performed using (BN prospec, Siemens, Erlangen, Germany), where the serum samples were collected from liver cirrhosis patients, healthy normal individuals, and analyzed for alpha 1 antitrypsin enzyme. The analysis was carried out as per the instructions on the manual. Alpha 1 antitrypsin was measured after appropriate dilution of the serum sample in diluent buffer. The diluted sample was mixed with antibody specific for alpha 1 antitrypsin. Antigen antibody complexes were formed after the reaction. A light beam having a wavelength of 840 nm was passed through a solution containing antigen antibody complex and the light scattered by the antigen antibody complex was measured. The amount of light scattered was proportional to the amount of antigen antibody complex in the solution. The normal reference range for this test is from 120 to 200 mg/dL. Routine laboratory techniques were used to detect biochemical parameters.

Phenotyping was carried out using isoelectric focusing (IEF) on 5% polyacrylamide gels at a pH of 4–5; samples were electrophoresed, and fixed before they were stained with Coomassie Brilliant Blue. AAT phenotypes were defined by visual inspection and compared with known patterns.

The QIAamp DNA Mini and Blood Mini kit from Qiagen was used to perform DNA extraction, according to the manufacturer's instructions. Genotyping was defined with the real-time PCR procedure used in Kaczor et al.^[14]^ In this reaction, 4 oligonucleotides were used, along with a pair of PCR primers and a pair of dual-labeled allele-specific fluorescent probes. Synthesis of primers and probes were carried out by Qiagen, with the sequence per the protocol in Kaczor et al.^[14]^

Two sets of probes are labeled complementary to the flanking sequence of the mutations were labeled at the 5 prime end with the reporter dye and at the 3 prime end with a quencher (Black Hole Quencher). The probes differed only by the 1 variable nucleotide for the PIZ allele reaction. The probes for a wild-type sequence in the PIS reaction were shifted by 3 nucleotides upstream and shortened by 3 bases to progress the specificity of the assay. The variable nucleotides for each of the sets of probes were near the 3 prime ends. Probes for the wild-type were labeled with 6-carboxy-X-rhodamine (ROX), but mutant alleles were labeled with 6-carboxyfluorescein (FAM) Table 2 as per Kaczor et al.^[14]^

**Table 2:**
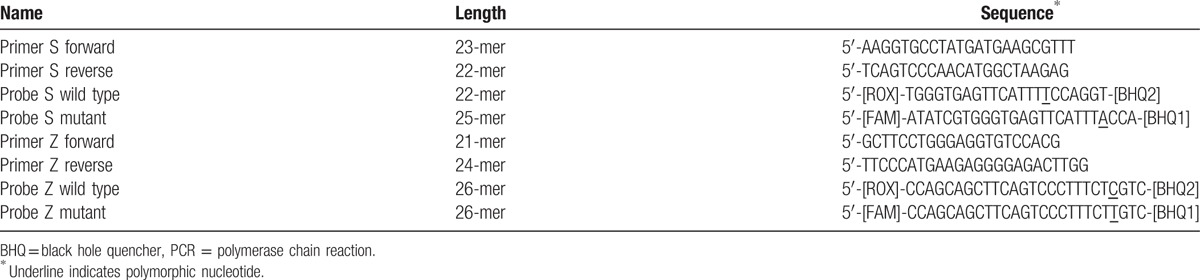
Primers and probes sequences for real-time qualitative PCR assay, as per Kaczor et al^[14]^.

### 2.1 View it in a separate window

The real-time allelic discrimination assay, which was based on the 5 prime nuclease activity of *Taq* polymerase, was performed in a total volume of 50 μL containing 3 μL of the genomic DNA template, 1 U of *Taq*polymerase (Finnzymes), 20 pmol of each primer (TIBMOLBIOL), 2 pmol of each probe (IDT DNA, Coralville, IA),10 nmol of each dNTP (Fermentas), and 2% dimethyl sulfoxide (Sigma, St. Louis, MO) in a buffer composed of 15 mmol/L ammonium sulfate, 60 mmol/L Tris HCL, pH 8.9, 3.5 mmol/L magnesium chloride, 0.02% Tween 80 and 0.002% 2-mercaptoethanol.

Amplification was carried out on a thermocycler with optical module (iCycleriQ; Bio-Rad, Hercules, CA). Fluorescence data were collected for each cycle at the end of annealing/extension using appropriate filter sets, including excitation/emission of 490/530 nm for FAM and 575/620 nm for ROX. Experiments were conducted in a 96-well plate. The genotyping results were analyzed using the iCycleriQ Optical System Software v.3 (Bio- Rad).

The phenotyping and genotyping results were compared and PCR products were sequenced for discordant samples, with a big dye sequencing kit (ABI, CA). Each exon was sequenced in a Genetic Analyzer (ABI, 310) and the genotypes of SERPINA1 gene were analyzed using the DNA analyzer (Applied Bio systems, CA) and Big Dye terminator sequencing. The sequence of the genotypic variants from the analysis was fed into software (codon code Aligner), and the genetic variations were noted.

### 2.2 Statistical analysis

SPSS Pc+ version 21.0 statistical software was used to analyze the data. Descriptive statistics (mean, standard deviation, frequencies and percentages) were used to describe the quantitative and categorical variables. The comparison of mean values of quantitative variables between men and women subjects (cases) was carried using a Student's *t*-test. Pearson's chi-square test was used to compare the distribution categorical variables between men and women subjects (cases). A Hardy–Weinberg equilibrium chi-square test was used to quantify the deviation of genotypes and allele in cases and controls. The statistically significance of the results was determined using a *P*-value of <0.05.

## 3 Results

**Figure 1:**
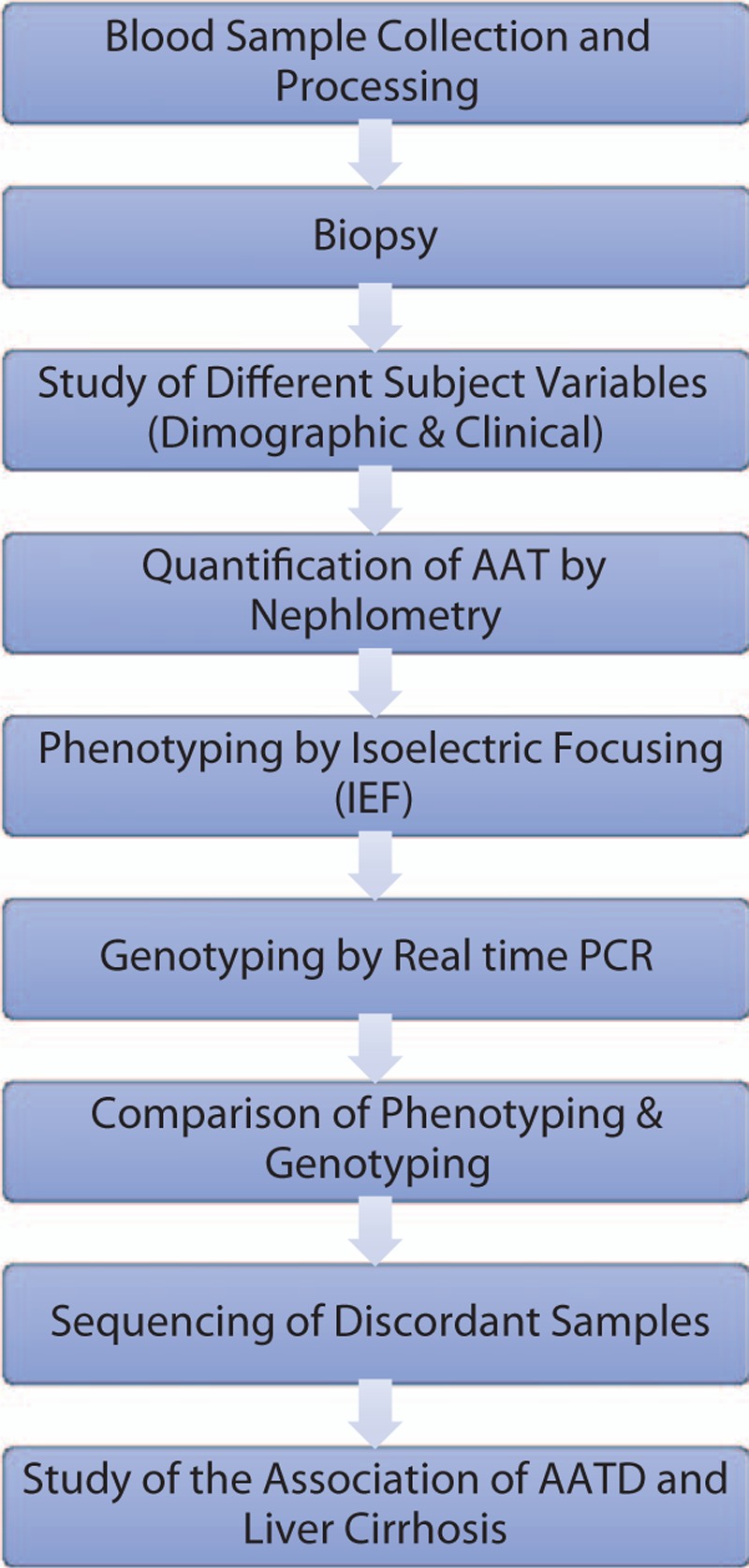
Patient flow diagram.

Overall, 400 eligible subjects were identified at the beginning of the samples collection, and 100 were excluded due to, insufficient sample amount (20), no informed consent (50) or erroneous diagnosis (30). The other 300 patients complete each stage of the study with full data. Figure 1 illustrates the diagnostic stages of the patients.

Phenotyping was carried out with isoelectric focusing gel electrophoresis to detect AAT phenotypes. Genotyping was designed to identify the most common deficiency alleles (Z and S). If neither the Z nor the S allele was detected, we used the terms non-S and non-Z in the results.

A comparison of phenotypes and genotyping results for liver cirrhosis patients and controls can be seen in (Table 3). In the control group, 70% of subjects were had “MM” phenotypes, 18.5% had “MS” and 10.5% had an “SS” phenotype, and for 2 samples (0.5%), 1 was FM, and the other FF. From the interpretation of the genotype assay with respect to the Z and S alleles, 284 of the 400 controls samples had the homozygous non-S, non-Z genotype, 74 were heterozygous (non-S, non-Z) S, and 42 were homozygous SS. The genotype and phenotype results were 99% concordant where 4 cases were discordant. In the first 2 cases, the phenotype result of FM was discordant with an MM genotype result, but the subsequent phenotype assay provide a result of MM for the first 2 samples, which was consistent with the genotype result and indicated that a phenotyping error had occurred. The other 2 cases were originally phenotyped as FF, but both of them were genotyped as MM. We could not repeat the phenotyping for these 2 samples due to low amount of available serum and poor sample quality, but no variation was detected for these samples in gene sequencing and this outcome agreed with the genotype and with the concentration of the AAT where it was 2.4 and 2.1 g/L.

**Table 3:**
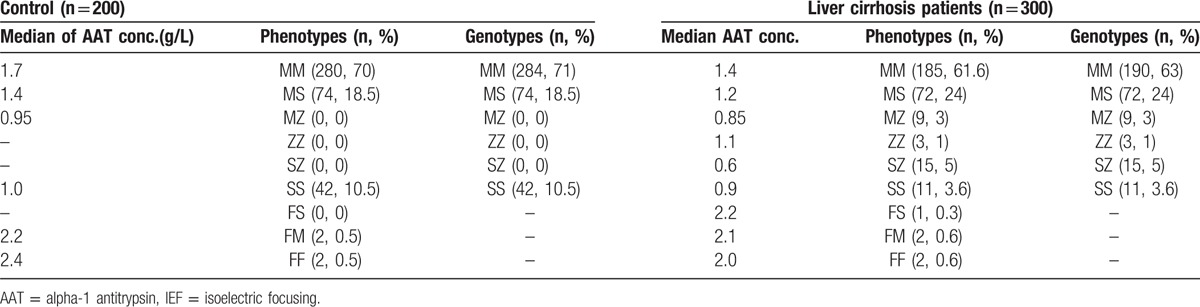
Comparison of IEF phenotypes and genotyping results for liver cirrhosis patients and controls.

Among 300 liver cirrhosis patient samples that were collected, 61.6% of subjects had “MM” phenotypes, 24% of them had “MS,” 3% had an “MZ” type, 1% had ZZ, 5% SZ, 3.6 SS, and 0.3, 0.6, 0.6 were had the types FS, FM and FF, respectively. In the genotyping results, 190 samples were homozygous with a non-S, non- Z genotype, 72 were heterozygous (non-S, non-Z) S, 9 were heterozygous (non-S, non-Z) Z, 3 were homozygous ZZ, 15 were heterozygous SZ, and 11 were homozygous SS. Five cases were discordant, including 2 cases that were FM, 2 that were FF and 1 case with FS phenotypes, that were all the MM genotype. The repeated phenotype tests were MM for the 2 FM and the 2 FF phenotypes samples, and no variation was detected in these samples using gene sequencing. The last sample was phenotyped as FS and genotyped as MM, and the subsequent phenotype assay confirmed the first result (FS), but no variation was detected using gene sequencing. The AAT quantification was 2.2 g/L, which was consistent with the MM genotype, and the reason for the discrepancy between the phenotype and genotype for this case remains unknown.

Table 4 shows the demographic and clinical data for study subjects with liver cirrhosis, and there was a highly statistically significant difference in the mean values of age, AST, ALT, AP, GGT, billiburin, and serum albumin related to the gender of the liver cirrhosis patients. The mean age of the female patients was significantly higher than the mean age of the male patients. However, the mean values of AST, ALT, AP, GGT, total bilirubin, and serum albumin values of the male patients were significantly higher than the mean values of the female patients. Additionally, there was no significant difference in the mean values of AAT concentration or prothrombin time of male and female patients.

**Table 4:**
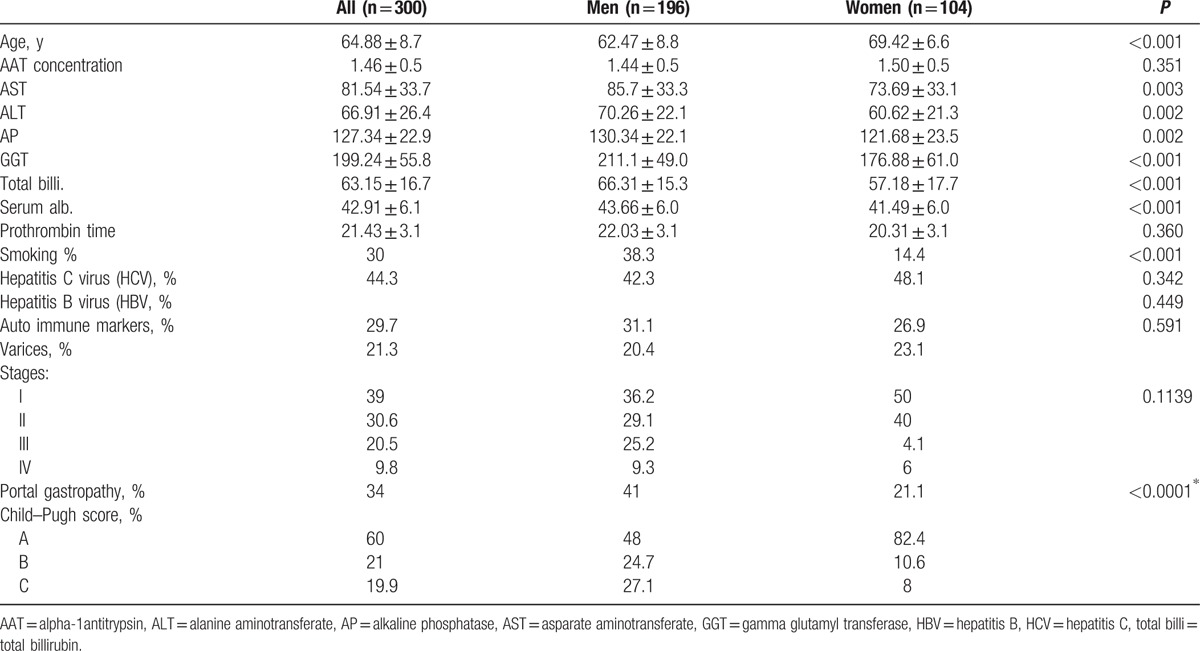
Distribution and comparison of mean values of study variables (demographic and clinical) of study subjects with liver cirrhosis.

Furthermore, there was a statistically significant difference between male and female patients regarding their smoking habits in that a higher proportion of males than females were smokers. There was no significant difference between male and female liver cirrhosis patients in terms of the proportion of hepatitis C virus, hepatitis B virus, and auto-immune markers.

The same table shows the varices percentages, where the women percentages of stages I and II were more than that for male and the opposite is true for stages III and IV in men, but the differences between numbers of varices stages between men and women were not statistically significant. Portal gastropathy percentages were higher in men than in females, and their numbers were statistically significant. In Child–Pugh score, Table 4 shows higher percentage for score A in women than in men, and the opposite is true for scores B and C.

Table 5 represents the distribution of the patients group and controls genotypes. We noticed that the distribution of the genotypes of liver cirrhosis patients indicated statistically significant deviations from Hardy–Weinberg equilibrium for 3 alleles (M,Z & S) of 6 genotypes (MM, MS, SS, MZ, ZZ, and SZ).

**Table 5:**
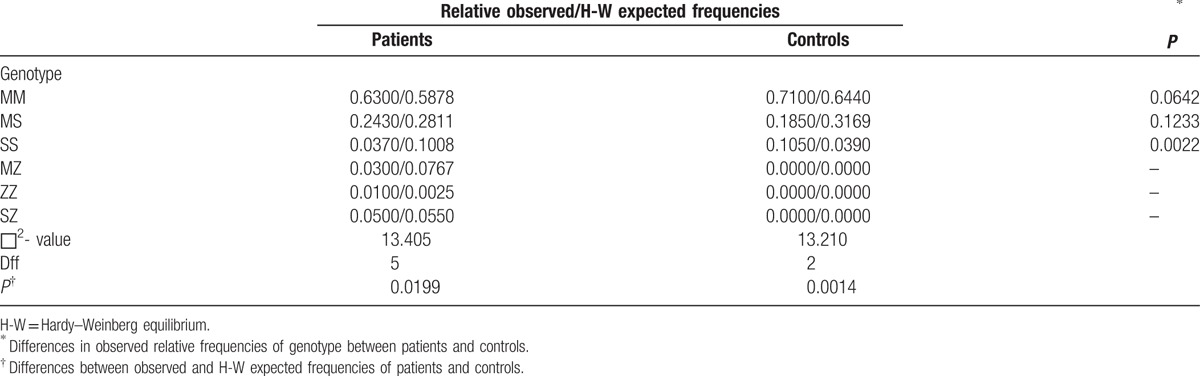
Distribution and comparison of relative observed/H-W expected frequencies of genotypes in liver Cirrhosis patients and healthy controls.

For the controls, as well, a statistically significant deviation from Hardy–Weinberg equilibrium was detected for 2 Alleles (M & S) of 3 genotypes (MM, MS, and SS).

The comparison of the distribution of (MM, MS, and SS) between liver cirrhosis patients and controls indicated there was a statistically significant difference in the observed frequencies, and the observed frequency of SS was significantly higher in healthy controls than in liver cirrhosis patients. There was no statistically significant difference in the observed allele frequencies (MM and MS) between liver cirrhosis patients and controls.

The results of Table 6 indicate that there was a highly statistically significant difference in the mean values of age, AAT concentration, AST, ALT, and prothrombin time of liver cirrhosis patients related to which patients were carriers of the Z allele. In other words, the mean age and AAT concentration of patients who were Z allele carriers is statistically significantly lower than those of the liver cirrhosis patients who were not Z allele carriers. Additionally, the mean values of AST, ALT, and prothrombin time of liver cirrhosis patients who were Z allele carriers were significantly higher than those in the liver cirrhosis patients who were not Z allele carries. No significant differences were found in the mean values of AP, GGT, total billirubin or the serum albumin of liver cirrhosis patient who were or were not Z allele carriers.

**Table 6:**
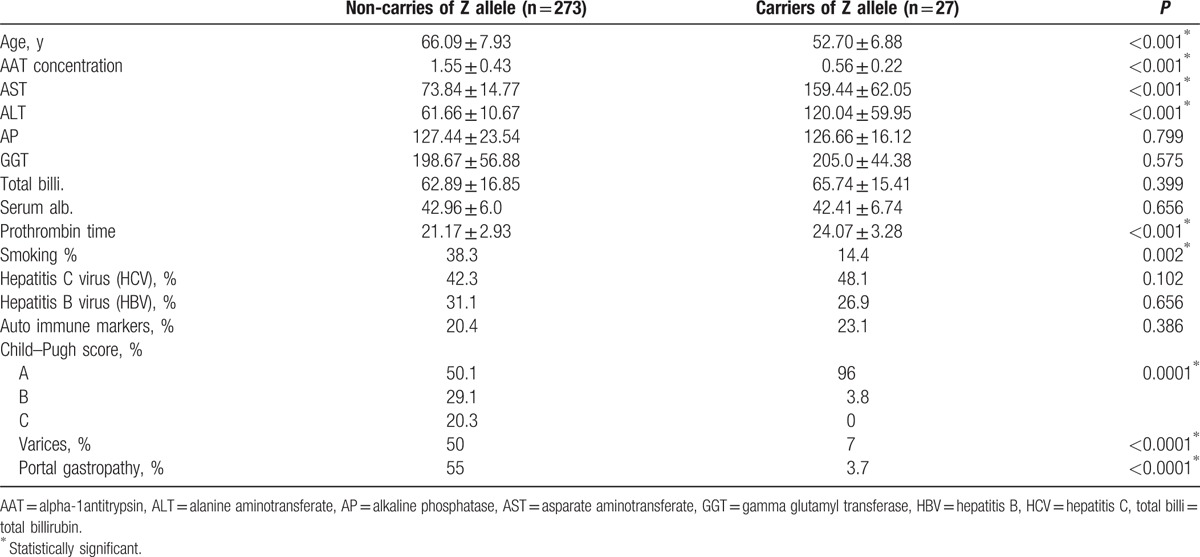
Distribution and comparison of the mean values of study variables (demographic and clinical) of liver cirrhosis patients according to the presence of the Z allele.

Additionally, there was a significant difference between liver cirrhosis patients who were or were not Z allele carries, related to their smoking habits. A higher proportion of patients who were not Z allele carriers were smokers compared to patients who were Z allele carriers. Additionally, there was no significant difference between patients who were or were not Z allele carriers related to the proportions of hepatitis C virus, hepatitis B virus, and auto immune markers that were detected. The majority of Child–Pugh scores in patients who carried Z allele were score A (96%), those patients were have less percentages of cases for varices and portal gatropathy compared to patients who do not have Z allele.

## 4 Discussion

Our exploratory determination of the association between liver cirrhosis with AATD in a Saudi population is of major importance because there was a lack of data about AAT deficiency. We found that adult patients who are homozygous AATD (ZZ) are at risk for developing liver cirrhosis. We must admit that there were some limitations to our research. The sample size must be larger because a large number of patients and healthy controls refused to participate in the research. Additionally, we could not support an association between liver cirrhosis and heterozygous Z alleles such as (MZ and SZ), because there were no alleles except for MM, MS, and SS variants in the healthy controls, which may have arose due the small number of available subjects in the kingdom.

In this study, we found that, the first step for AATD diagnosis must be, the determination of the serum AAT concentration using nephelometry, followed by genotyping using real-time PCR. This technique was very accurate and provided the correct results in 100% of cases. It was also faster and cheaper than phenotyping. Genotyping detected only the most common deficiency alleles, including Z and S. Thus, individuals with no S or Z variants and low AAT concentrations need phenotyping to acquire a definitive diagnosis due to this limitation of genotyping. Another limitation is that gene sequencing can be used to confirm discordant samples, but even though this technique can identify any mutation, is very expensive.

Quantitative determination of serum AAT level is not sufficient for the diagnosis of AAT deficiency because AAT is an acute phase reactant, AAT may be increased due to inflammation, which leads to misdiagnosis. Therefore, there is a need for effective testing such as phenotyping and genotyping to increase the detection of individuals who are at risk.^[10]^

The performance of genotyping and phenotyping in the diagnosis of AAT deficiency has been compared by several authors.^[15,16]^ Phenotyping of our samples resulted in more errors than did genotyping, so genotyping simplified the diagnosis of AAT deficiency. This outcome agreed with the results of Noel McElvaney,^[17]^ who noted that phenotyping is the current gold standard for detecting many AATD variants (except null variants), while advances in molecular techniques have facilitated a better diagnosis and have made genotyping more effective.

Liver disease caused by AATD in childhood has been well documented, but the association between liver disease and AATD in adults is less clear. It is difficult to determine the risk of cirrhosis in adults because the majority of data are retrospective and taken from individuals with AAT-deficient lung disease or cirrhosis.^[18,19]^ Some epidemiological research studies^[20]^ have stated that infants with Pi ZZ suffer from clinical signs of neonatal cholestasis and other clinical symptoms of liver disease without jaundice. In adults, Kok et al^[21]^ demonstrated that Pi ZZ- carrying adults have a higher risk of developing end-stage liver disease, cirrhosis, and hepatocellular carcinoma. Additionally, they found that heterozygosity for AATD was co-factor causing chronic liver disease, and it can affect the course of hepatitis C, hepatocellular carcinoma, end-stage liver disease and cirrhosis, clarifying of the relation between heterozygosity for AATD and liver disease in the adults is of clinical importance.

In this research, a higher frequency of Pi ZZ homozygosity than expected was found among patients with liver disease, which caused deviation from the Hardy–Weinberg equilibrium and confirmed a previously suggested correlation between severe liver disease and the Pi ZZ genotype.

An increased risk of cirrhosis and liver cancer in Pi ZZ patients was found in a retrospective study.^[22]^ Male Pi ZZ homozygous patients have a higher risk of cirrhosis and HCC,^[23]^ independently of hepatitis B or C infection. Our results in the study group supported these findings. However, we failed to demonstrate a different frequency of Pi ZZ homozygosity between patients with liver cirrhosis and healthy individuals, which may be due to the absence of Pi ZZ in the control samples.

There have been different views about the role of the Z allele in heterozygous AATD patients in the pathogenesis of chronic liver disease. Some studies have established some evidence of a relationship between the heterozygous Z allele alpha-1-antitrypsin phenotype and end-stage liver disease with different etiology.^[24,25]^ An association between Pi MZ and liver disease was demonstrated using 1055 liver biopsies that were screened for AAT depositions in hepatocytes. Overall 34 patients with these inclusions were phenotyped and the percentage of Pi MZ phenotype in the whole biopsy group was 2.4%, whereas 9% of liver cirrhosis patients had aPi MZ phenotype. A prevalence of 21% Pi MZ was observed in cryptogenic cirrhosis and in chronic active hepatitis, and the percentage was significantly increased relative to other causes of cirrhosis. The prediction of the Pi MZ cirrhotic patients was poor, because most patients died within 1 year.^[26]^

Patients with end-stage liver disease, who needed liver transplant, were investigated. Pi MZ was found in 7.3 to 8.2% of patients compared to 2.8% in the control population. A heterozygous phenotype was more prevalent in patients with hepatitis C, alcoholic liver disease, cryptogenic cirrhosis, and hepatocellular carcinoma.^[27]^ Furthermore, 3 patients had a rapidly deteriorating clinical course and alcoholic liver disease, which lead to their’ death. The 3 patients had heterozygous phenotypes.^[28]^ However, others researchers have failed to found any association between heterozygous MZ AATD and cirrhosis.^[29,30]^ These studies showed that various liver diseases affected the AAT deposits and AAT deposition affected the course of the liver disease. In our study, we were not able to show differences in Pi MZ and Pi SZ frequency between patients and controls because the only variants in healthy individuals were MM, MS, and SS. Regarding Pi MS, there was no statistically significant difference in its frequency between liver cirrhosis patients and controls.

Other authors^[31]^ have shown that the onset of liver disease symptoms starts at 58 years of age in Pi ZZ, 66 years in Pi SZ, and 73 years in Pi MZ carriers. The age of our Pi ZZ carriers were 44, 55 years for Pi SZ, and 66 years for Pi MZ.

In patients who were Z allele carriers, the mean age and AAT concentration significantly lower than in the liver cirrhosis patients who were not Z allele carriers, and some studies such as^[24]^ suggested that AATD may be a risk factor for infected HBV individuals that develops from the carrier stage to chronic and cirrhotic stages. Male gender and obesity, but not alcohol or viral hepatitis, were associated with predisposition to advanced liver disease in adults with AATD.^[32]^ This trend clearly appeared in our research because the mean age of patients with liver cirrhosis was 62.47 ± 8.8 for men and was 69.42 ± 6.6 for women. Additionnally, the AAT concentrations were 1.44 ± 0.5 and 1.50 ± 0.5 in men and women respectively.

Topic et al^[23]^ stated that there were no differences in the activity of liver enzymes in patients relative to the presence of the Z allele, and our data showed that the mean values of AST, ALT, and prothrombin time of liver cirrhosis patients who were Z allele carriers were significantly higher than those in the liver cirrhosis patients who were not Z allele carries. However, there was no significant difference in the mean values of AP, GGT, total billi, and serum albumin of liver cirrhosis patient who were or were not and without Z allele carriers.

It is not known why AAT deficiency leads to lung disease in some patients and liver disease in others. There is some evidence that environmental factors are responsible for this difference. Pulmonary disease appears in homozygous Pi ZZ individuals who are smokers or who are have been exposed to air irritants.^[33]^ It has been shown that smoking is a risk factor for pulmonary disease because AATD smokers develop emphysema at a younger age; however, nonsmokers are at risk for liver diseases that manifest later in life, and 32% to 37% of AAT deficient nonsmoking patients will die as a result of AATD-induced liver disease.^[33]^ In our data, there was a statistically significant difference between liver cirrhosis patients who were or were not Z allele carries that was related to their smoking habits. A higher proportion of patients who were not Z allele carriers were smokers compared to patients who were Z allele carriers, which indicated that smoking may also be a risk factor for liver diseases. Apart from environmental factors, there is some evidence that genetic factors can modify the risk for AATD-related end-organ damage.^[34]^

In this study, we found no significant difference between patients who were or were not Z allele carriers in relative to the proportion of hepatitis C virus, hepatitis B virus, and auto-immune markers found in patients. This result agreed with the results of Topic et al^[23]^ and partially agreed with the results of Stoller and Aboussouan^[35]^ who stated that the hepatitis C virus is an exogenous risk factor for chronic liver disease in Z alleles carriers, whereas autoimmunity, alcohol abuse, and hepatitis B virus were not risk factors. Our study results also disagree with some reports that stated that Z and S allele heterozygosity were associated with (end-stage) liver disease due to HCV, alcoholic liver disease, and cryptogenic cirrhosis.^[21,24]^

## 5 Conclusion

It is important to use genotyping in addition to AAT concentration to detect AATD because the serum protein level alone is not sufficient for accurate diagnosis to help physicians to intervene with measures such as quitting smoking and augmentation therapy. There is a need for programs to educate therapists about AATD for early diagnosis and increase the chances of discovering people with AATD. Adult patients who are homozygous AATD (ZZ) are at risk for developing liver cirrhosis, whereas more research studies are needed to determine the effects of heterozygous AAT deficiency on the liver. Additionally, males were more susceptible to liver cirrhosis than females.
